# The Current Evidence and How-To on Combined Sacrocolpopexy and Rectopexy

**DOI:** 10.1007/s00192-024-05869-2

**Published:** 2024-08-01

**Authors:** Amy D. Gee, Sunny Kairi Lee, Kristen Ban, Marie Fidela R. Paraiso

**Affiliations:** 1 Department of Urogynecology, Cleveland Clinic, 9500 Euclid Ave, A81, Cleveland, OH 44195 USA; 2 Department of Colorectal Surgery, Cleveland Clinic, Cleveland, OH USA

**Keywords:** Combined sacrocolpopexy and rectopexy, Defecatory dysfunction, Multicompartment prolapse

## Abstract

**Introduction and Hypothesis:**

Multicompartment prolapse is a complex pelvic floor condition that can involve coordination of both urogynecologists and colorectal surgeons.

**Methods:**

Concomitant sacrocolpopexy and ventral rectopexy is a safe and effective approach to treating this condition.

**Results:**

The combined procedure has high rates of patient satisfaction and symptom improvement, including defecatory dysfunction, sexual health, and overall increased quality of life.

**Conclusion:**

Concomitant sacrocolpopexy with ventral rectopexy is safe and improves overall outcomes.

## Introduction

Pelvic organ prolapse is a complex condition involving the herniation of pelvic organs due to the loss of supportive structures. It is difficult to accurately estimate the prevalence of pelvic organ prolapse owing to the wide variety of reported symptoms and lack of medical attention to those affected. In the USA, the prevalence of pelvic organ prolapse is estimated to range from 2.9% to 8.0% [1, 2]. Pelvic organ prolapse can be further complicated by concomitant rectal prolapse, which can occur in up to 34% of cases [3]. There is a growing need for a multidisciplinary approach to treating multicompartment prolapse, evidenced by the increased rate of combined sacrocolpopexy and rectopexy procedures from 2.6% to 7.7% between 2005 and 2014 [4].

## Outcomes Following Combined Sacrocolpopexy and Rectopexy

A combined approach to the treatment of rectal and vaginal prolapse is preferred for several reasons. Patients who have undergone the combined surgery report greater satisfaction, including improved defecatory function, increased quality of life [5, 6], less symptom bother, better sexual function, and improved overall quality of life [7]. For patients with multicompartment prolapse, failure to address and reconstruct all compartments is partly responsible for high surgical failure rates, reported to be as high as 58% when only a single compartment is repaired [8]. Reoperation rates reach as high as 33% in patients who require a procedure for prolapse in a secondary compartment [9, 10].

The safety of combined ventral mesh rectopexy and sacrocolpopexy has been evaluated. A National Surgical Quality Improvement Program (NSQIP) database was utilized to identify 206 women who underwent combined rectopexy and sacrocolpopexy between 2005 and 2014. These 206 cases were compared with 3,394 cases of women who underwent isolated rectopexy. Morbidity was not significantly different in the two groups (14.8% rectopexy vs 13.6% rectopexy plus sacrocolpopexy, *p* = 0.65) [4]. A later NSQIP study from 2013 to 2016, showed an overall complication rate of 8.9% for combined procedures. Complication rates between sacrocolpopexy, rectopexy, and combined procedures were not significantly different (6.2 vs 7.6 vs 8.9%; *p* = 0.058) [11]. Watadani et al. performed a retrospective review of 110 women who underwent combined rectopexy and sacrocolpopexy. Fifty-two patients completed the follow-up questionnaires. There was significant improvement in post-operative constipation, with 82% of patients reporting symptom resolution. Fecal incontinence severity scores also improved post-operatively and 82% of incontinent patients reported cure or improvement [6].

Aside from improved patient outcomes, there are numerous additional benefits to this combined surgical approach, including safety and cost-effectiveness [5, 12–14]. By providing comprehensive pelvic floor reconstruction with a single operation, there is also reduced anesthesia risk, hospital stay, and post-surgical recovery [15]. Although the combined surgery increases the duration of operative time, this additional operative time is minimal owing to similar pelvic dissections required for the operation [16]. Furthermore, multiple studies have demonstrated that there is no increased risk of complications or post-operative health care resource utilization of the combined approach compared with sacrocolpopexy or rectopexy alone [12, 15, 17].

## Route of Combined Sacrocolpopexy and Rectopexy

Both pelvic organ prolapse and rectal prolapse can be surgically treated via transabdominal or transperineal routes. The transperineal approach is generally reserved for medically and surgically high-risk patients including those with suspected abdominal adhesions from multiple surgeries and/or those who cannot tolerate several hours of the Trendelenburg position [14, 18–20]. If possible, a minimally invasive, transabdominal approach using laparoscopy or robotics-assisted laparoscopy is preferred. Lower complication rates and decreased hospitalization have been shown to be associated with combined minimally invasive sacrocolpopexy and rectopexy compared with open surgery [21, 22].

Regarding rectal prolapse repair, the five main approaches are as follows: transabdominal rectopexy with or without sigmoid resection, perineal rectosigmoidectomy (Altemeier procedure), perineal mucosal stripping and muscular plication for rectal prolapse (Delorme procedure), and ventral mesh rectopexy. An anterior approach to rectal mobilization is preferred over a posterior approach, as data suggest improved bowel function outcomes [23]. Both abdominal sacrocolpopexy and ventral rectopexy are considered gold standard procedures for vaginal apex and rectal prolapse respectively compared with the transperineal approach owing to improved structural and functional outcomes and reduced risk of recurrence [5, 16, 24–27]. In this article, we focus on robotics-assisted ventral mesh rectopexy with combined sacrocolpopexy for the surgical treatment of multicompartment pelvic organ prolapse.

## Operative Approach to Combined Sacrocolpopexy and Ventral Mesh Rectopexy

Organizing and coordinating combined surgeries can create challenges when scheduling patients. If feasible, a combined colorectal and urogynecology clinic should be organized in which both surgeons are present to counsel and organize a surgical plan. One combined clinic a month may be enough for most surgeons, depending on volume. If a patient is seen in a urogynecology or colorectal clinic with concerns for multicompartment prolapse, a message is typically sent through the electronic medical record to the surgeon in charge of the clinic. Additional testing can be ordered, such as defecography, anal manometry, bowel transit study, or urodynamic evaluation in preparation for the upcoming combined colorectal/urogynecology clinic appointment. Manometry can identify the etiology of rectal prolapse, whether tissue laxity or pelvic floor nonrelaxation. This information helps providers to understand if pelvic floor physical therapy will improve the post-operative outcome and can guide education regarding post-operative functional outcomes. Educating administrative assistants on this process or assigning one to coordinate combined appointments and surgeries is very helpful. Rotating cases in different specialty operating rooms can expedite efficiency and ultimately allow more patients to undergo combined surgeries. Often, two combined surgeries will be scheduled with an early morning start time. One case will start in the colorectal surgeon’s operating room and the other case will start in the urogynecologist’s operating room. The respective portions of the surgery can be performed and then surgeons can switch rooms to complete their portions. Ideally, two dedicated operating rooms side by side would allow completion of four combined abdominal sacrocolpopexy with ventral mesh rectopexy procedures in 1 day.

In the operating room, a trained robotics surgery team is preferred, and instruments should be checked and brought into the room before the case starts to increase efficiency. The patient is placed on a foam pad with a padded chest strap to prevent sliding during steep Trendelenburg placement. She is positioned in dorsal lithotomy with arms tucked and generously padded at all pressure points to prevent nerve injury. The patient’s buttocks are positioned a few inches off the edge of the operating table to allow for full manipulation of the vagina, uterus, and/or rectum. Examination under anesthesia of the vagina and rectum is performed by both surgeons and confirmation of the surgical plan is discussed. During rectal examination, stool is removed from the rectal vault digitally or with a mushroom tip catheter with betadine irrigation.

Once the patient is prepped and draped, a Foley catheter is inserted. If a uterus is present, a uterine manipulator is placed. Port placement is surgeon preference. Typically, an 8-mm vertical incision in the inferior border of the umbilicus is made. This type of incision allows for easy extension for removal of the uterine corpus if a supracervical hysterectomy is performed. Pneumoperitoneum can be achieved with the Veress needle, Hasson open technique, or with an optical trocar. The 8-mm trocars are placed under direct visualization about 8–9 cm apart in a straight, horizontal line from the umbilical port. Two ports are placed on the left side of the umbilical port, at least 8 cm apart. Two additional ports are placed on the patient’s right side of the umbilicus, at least 8 cm apart. The furthest right, lateral port is the assist port. This can be placed in the patient's right lower quadrant, careful to avoid the superficial and inferior epigastric vessels. In summary, port placement consists of the following: 8-mm umbilical camera port, three 8-mm robotics ports, and an 8-mm assist port in the right lower quadrant. The robot is docked and targeted to the pelvis. The following instruments are loaded into the robotic trocars: arm 1 (bowel grasper), arm 2 (fenestrated bipolar forceps or Cadiere forceps), arm 3 (8 mm 30° robotics laparoscope), and arm 4 (monopolar scissors). A robotics needle driver and suture cut needle driver are in the room for mesh attachment. The patient is then placed in steep Trendelenburg position to aid in bowel retraction for optimal visualization of the pelvis.

If a uterus is present, the hysterectomy can be performed first. If a hysteropexy is being performed, a Hulka or preferred manipulator can be placed to manipulate the uterus during anterior and posterior vaginal dissections. The bowels are retracted out of the pelvis with the robotic bowel grasper. The bedside assistant can also aid in bowel retraction out of the pelvis. A T’lift (Promecon, Hamburg, Germany), Endoloop (Johnson & Johnson, New Brunswick, NJ, USA), or suture can be placed through a sigmoid epiploica and externalized through the abdominal wall with a suture-grasping device and secured to aid in further bowel retraction if needed. The sacral promontory and right ureter are identified. Given the lack of tactile feedback with the robot, the bedside assistant can also aid in identifying the sacral promontory. The sacral dissection is performed either by the colorectal team or the urogynecology team. The camera should be turned to 30° looking down at the promontory. The promontory peritoneal incision is commenced midway between the rectal mesentery and right ureter. Robotic arm 1 (bowel grasper) should be used to sweep the sigmoid colon and its mesentery gently to the patient’s left. Using the grasper in robotic arm 2, the peritoneum overlying the sacral promontory should be elevated toward the anterior abdominal wall and electrosurgery with the monopolar scissors in arm 4 should be used to open the peritoneum. Once a peritoneal window is created, blunt dissection and careful sharp dissection are used to help identify the anterior longitudinal ligament. The tissue-spreading technique uses a blunt grasper to spread tissue caudal and cephalad and then uses the flat side of the monopolar scissors to sweep tissue laterally between the grasper tip. A skilled bedside assistant can also use a blunt laparoscopic Kittner to gently sweep tissue off the anterior longitudinal ligament to decrease the risk of bleeding in the pre-sacral space.

The peritoneal flaps are created to allow for the mesh to be covered at the end of the procedure. We prefer this technique over a tunneling technique that often used with sacrocolpopexy alone owing to the extensive rectal dissection and mesh attachment deep into the pelvis. Opening the peritoneum should be performed with monopolar scissors, taking care to dissect the underlying fat off the peritoneum before cutting. The dissection starts at the sacral promontory, bisecting the distance between the rectosigmoid and the right ureter, and continues caudally, just medial to the right ureter but lateral to the rectal mesentery. If the dissection becomes thick and significant adipose tissue is encountered, the dissection might be carried too far medially into the rectal mesentery. The right ureter is kept in view during this dissection. The dissection is continued all the way down to the posterior cul-de-sac until the right uterosacral ligament is encountered. The uterosacral ligament is usually dense tissue, so electrosurgery in this area is used. With an end-to-end anastomosis (EEA) sizer or other preferred vaginal manipulator in the vagina, the dissection is continued into the rectovaginal space. Often, an EEA sizer or alternative vaginal manipulator is placed in the vagina and, if indicated, into the rectum during this time, to maintain the proper plane of dissection.

The robotics camera should be rotated to look 30º upward to perform the dissection of the rectovaginal space caudal to the perineal body. Blunt and sharp dissection is used to open the rectovaginal space, careful to stay close to the EEA sizer that is in the vagina. Once this plane is developed, the colorectal or urogynecology surgeon continues the dissection caudally until bilateral levator ani muscles and the perineal body can be visualized. The vaginal sizer is angled towards the pubic bone whereas the rectal sizer is angled downward toward the sacrum. An Ally II Uterine Positioning System (Cooper, Trumbell, CN, USA) may be used if no vaginal assistant is available. The assistant between the legs can push on the perineal body and feel the robotic grasper, confirming the proper depth of dissection. The mesentery of the rectum is excised and the lead point of the rectal prolapse or intussusception is identified. The lead point can be identified by visualizing the most dependent or redundant portion of the rectum. Reviewing defecography images can be useful in correlating intraoperative findings with the lead point of the prolapse.

The urogynecologist then performs the anterior vaginal dissection. If a supracervical or total hysterectomy is performed, the plane from the bladder flap is continued to the level of the bladder trigone during the hysterectomy for procedure efficiency. An EEA sizer placed in the vagina is helpful for the anterior dissection. Depending on the patient’s anatomy, a Deaver or Breisky retractor instead of an EEA sizer can help to visualize the vesicovaginal space. The bladder is dissected off the anterior vagina until the trigone is reached. The Foley balloon in the bladder helps to identify the trigone. Typically, this distance is at least 5–6 cm. The bladder can be backfilled with normal saline to help to delineate the plane between the vagina and bladder. Another tip for visualizing the plane is to use a 10-ml syringe with a spinal needle and injectable saline or lidocaine and inject above the cervix, from the vagina, to hydro-dissect the vesicovaginal space. A spinal needle or laparoscopic aspirator needle may also be used for hydrodissection. All these techniques help to delineate a scarred vesicovaginal plane.

**Fig. 1:**
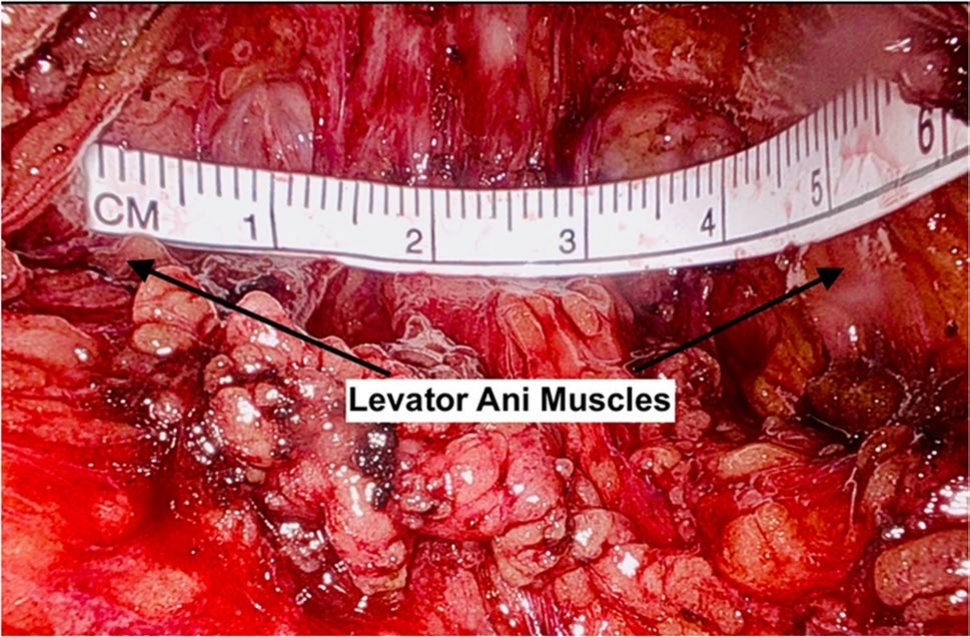
Small ruler measuring the distance between the levator ani muscles

**Fig. 2:**
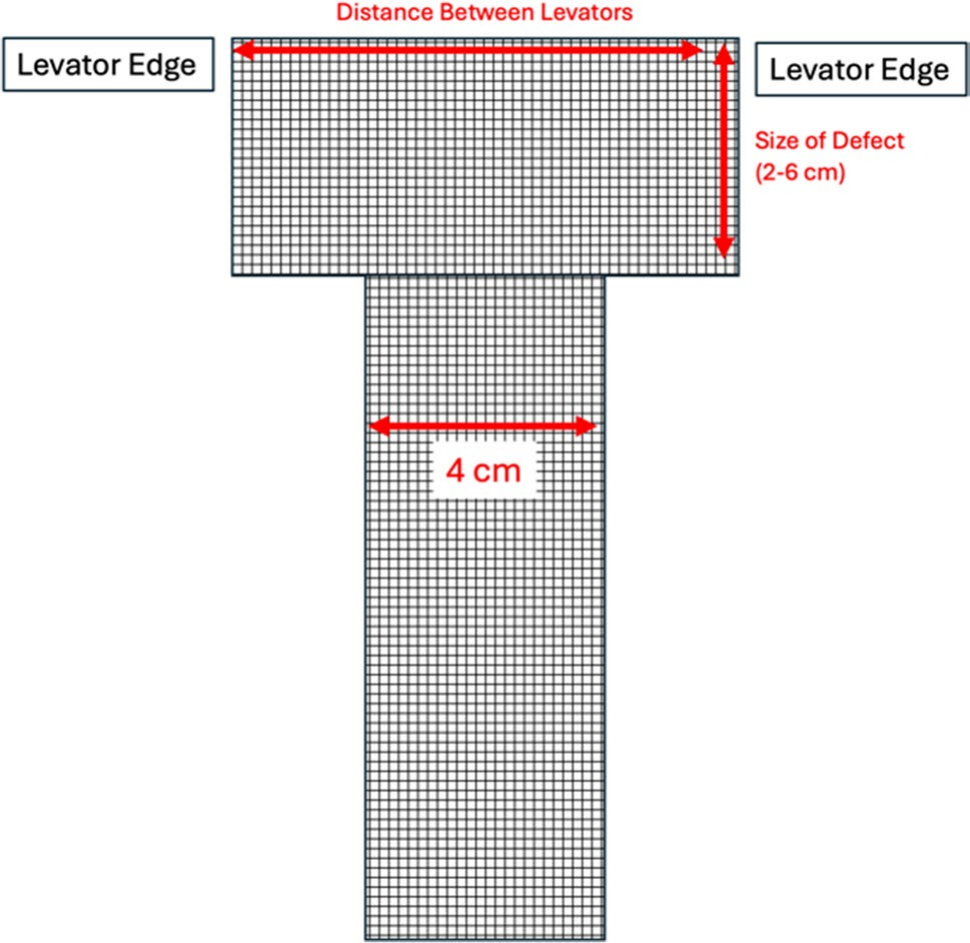
Mesh fashioned into a “T-shape” according to measurements taken intra-operatively

Once the sacral, anterior, and posterior dissections have been completed, the polypropylene mesh attachment can take place. Robotic arm 2 is exchanged for a needle driver and arm 4 is exchanged for a suture cut needle driver. A small ruler is placed into the abdomen and the distance between the levator ani muscles is measured (Fig. 1). This measurement is the inferior portion of the T-shaped mesh. A T-shape is fashioned from an 8 × 24 cm flat, type I polypropylene mesh sheet. The inferior portion of the T is the distance between the levator ani muscles. The lateral edges of the T are anywhere from 2 to 6 cm on each side, depending on the size of the defect (Fig. 2). The long portion of the T is about 4 cm in width. The total mesh length (24 cm) is kept. The T-shaped (or cross-shaped, if indicated) mesh is sutured to the bilateral levator ani muscles with delayed absorbable sutures, typically polyglactin 910; however, some surgeons may prefer Ethibond (Johnson & Johnson, New Brunswick, NJ). It is important to employ precise, shallow bites when suturing the mesh to the levator ani muscles to prevent post-operative discomfort. In patients with a pre-operative history of pain, it is advisable to avoid attachment to the levator ani muscles altogether. The mesh is then attached to the anterior surface of the rectum, taking seromuscular bites, with several 2–0 polydioxanone (PDS) or 2–0 polyglactin (Vicryl) sutures, until the mesh is flat on the rectum. During this step, an EEA sizer in the rectum can help to delineate the depth of the sutures and manipulates the rectum for suturing.

**Fig. 3:**
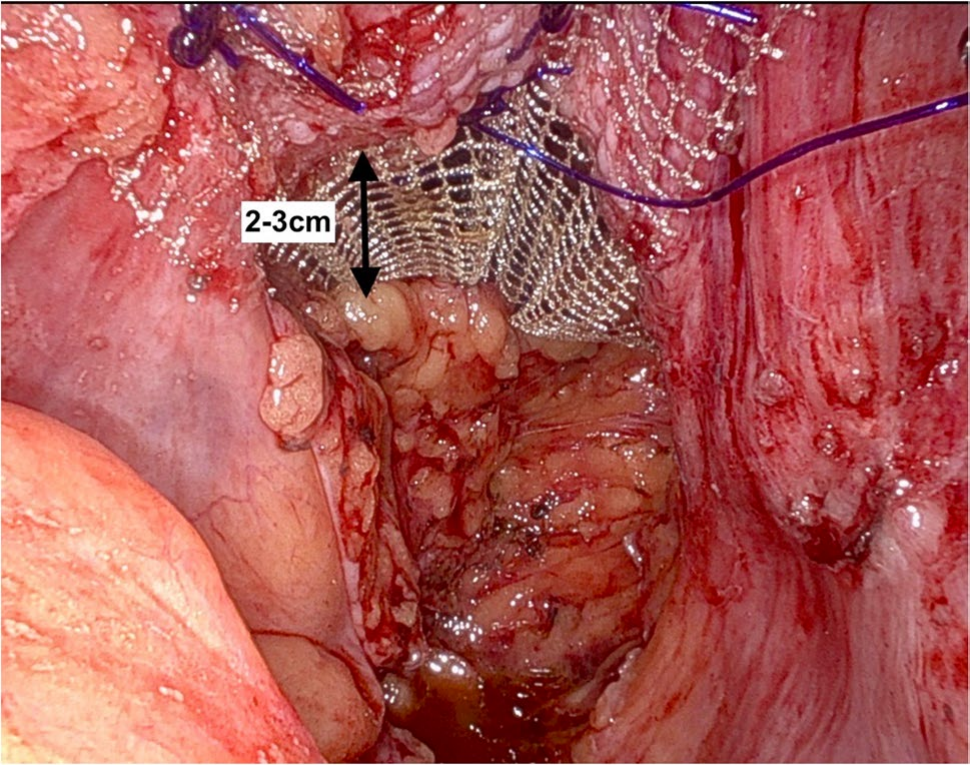
Distance between the rectal and vaginal mesh attachment is 2–3 cm

**Fig. 4:**
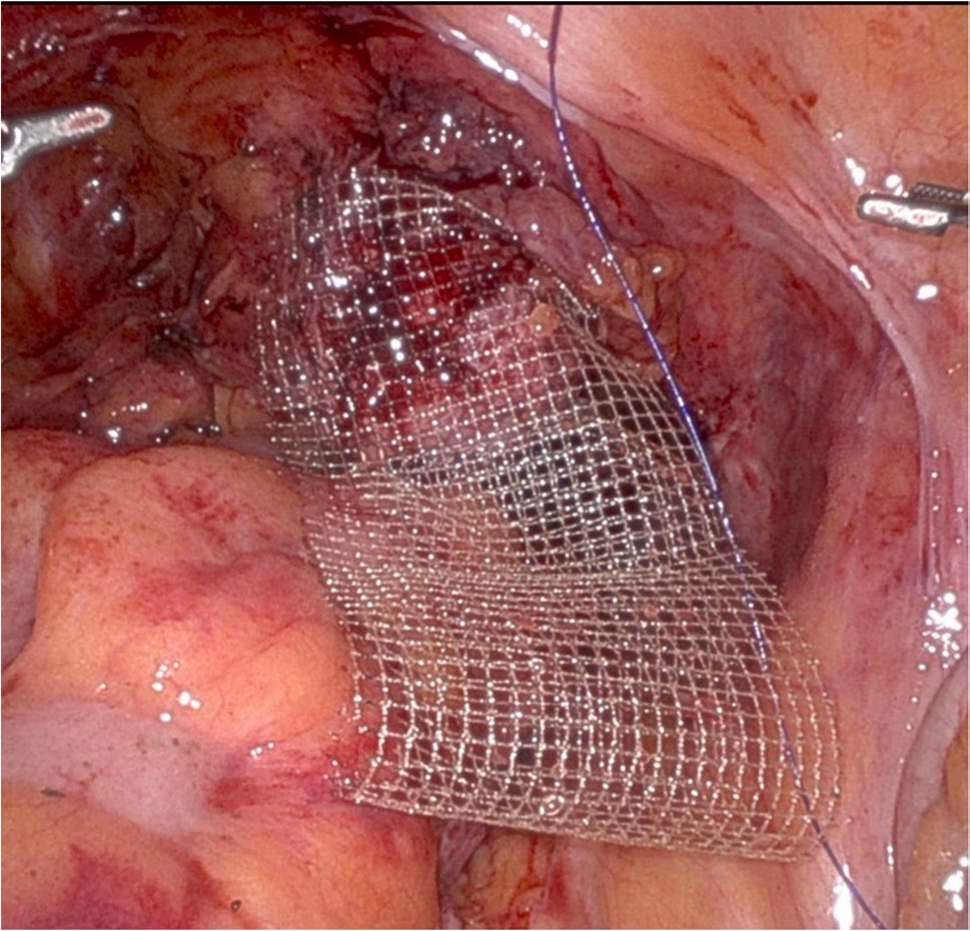
Double-layered mesh “tail” for sacral attachment

Once the mesh is secured to the anterior rectum, the urogynecology team sutures the mesh to the posterior and anterior vagina. Special attention is taken to ensure 2–3 cm between rectal and vaginal mesh attachment (Fig. 3). The same mesh attached to the rectum is sutured to the posterior vagina (approximately 2–3 cm from the top row of rectal sutures) with several 2–0 PDS sutures. This ensures that the posterior cul-de-sac is closed off, eliminating enterocele recurrence. The remainder of the mesh is folded over and sutured to the anterior vagina, leaving a double-layered mesh “tail” for suturing to the sacrum (Fig. 4). The mesh is tensioned so that the anterior, apical and posterior vaginal prolapse are elevated in a natural way without significant tension or stretch. The mesh tail is held to the sacrum with a grasper at the appropriate tension. A vaginal and rectal examination is performed to confirm appropriate tensioning. The mesh is then sutured to the sacrum with 2–3 sutures of 0-polypropylene suture. If the length of one piece of flat mesh is insufficient or, if indicated, a separate full-length arm of flat mesh can be sutured to the anterior vagina to tension the anterior and posterior walls separately.

The peritoneum is closed over the mesh based on preference to decrease the risk of small bowel obstruction or adherence of sigmoid epiploica to the mesh. During this step, it is important to dissect the right ureter off the overlying peritoneal flap or take care to incorporate the edge of the peritoneum, as aggressive suture placement can lead to ureteral damage or kinking on the right edge of the peritoneal flap. Cystoscopy is performed at completion of the procedure to evaluate for suture or injury to the bladder and ensure that the ureters are not kinked.

Our surgical technique for the combined procedure is available through the IAPS/IUGA platform with the following link: https://academyofpelvicsurgery.com/2022/03/04/video-19-combined-robotic-ventral-rectopexy-and-sacrocolpopexy/

## Conclusion

Combined robotics-assisted ventral mesh rectopexy and sacrocolpopexy is the emerging gold standard procedure for patients with multicompartment prolapse. Providing care to these patients can be optimized by organizing a colorectal and urogynecology team that specializes in multicompartment prolapse. With the data available, robotics-assisted ventral mesh rectopexy with sacrocolpopexy is safe and improves overall outcomes. Establishing patient factors and treatment goals is important for successful surgical outcomes.

## Data Availability

The data that support the findings of this review are listed in the reference section and are available on digital biomedical repositories such as PubMed.
